# Improving productivity in rabbits by using some natural feed additives under hot environmental conditions — A review

**DOI:** 10.5713/ab.22.0354

**Published:** 2023-01-11

**Authors:** Magdy Abdelsalam, Moataz Fathi

**Affiliations:** 1 Department of Animal Production and Breeding, College of Agriculture and Veterinary Medicine, Qassim University, Al-Qassim 51452, Saudi Arabia; 2 Department of Animal Production, Faculty of Agriculture, Alexandria University, El-Shatby, Alexandria 21545, Egypt; 3 Department of Poultry Production, Faculty of Agriculture, Ain Shams University, Hadayek Shoubra 11241, Cairo, Egypt

**Keywords:** Eucalyptus, Feed Additive, Hot Condition, Moringa, Propolis, Yucca

## Abstract

Heat stress is a major challenge to animal production in tropical and subtropical climates. Rabbits suffer from heat stress more than farm animals because they have few sweat glands, and their bodies are covered with thick fur. Intensive farming relies on antibiotics as antimicrobials or growth promoters to increase animals’ productivity and health. However, the European Union and many countries have banned or restricted the use of antibiotics in animal feed for human health concerns. Several studies have found that replacing antibiotics in rabbit feed with natural plants or feed additives increases productivity and improves immune capacity, especially under heat stress conditions. Growth performance, immune response, gut microflora, and carcass yield may be increased in rabbits fed a diet supplemented with some natural plants and/or propolis. In this review article, we discuss and summarize the effects of some herbs and plant extracts as alternative feed additives on rabbit productivity, especially for those raised under hot ambient temperatures.

## INTRODUCTION

Global warming is the main problem facing the farm animal industry. In general, high environmental temperatures have a negative impact on animal performance. Rabbits are more sensitive to heat stress than most farm animal species because they have few sweat glands, and their body is covered with a thick fur [1–3]. Growth performance, reproductive aspects, immunity, and health condition dramatically deteriorate in rabbits raised under heat stress [3–6]. Additionally, heat stress has a negative effect on intestinal mucosa and microbiota in rabbits [5]. Inclusion of antibiotics in animal feeds has already been banned in many countries for their harmful effect on human health and food safety. Replacement of antibiotics with natural feed additives in rabbit feeding is a main concern for organic livestock farming. Many plants and their extracts as feed additives have been used in animal nutrition such as Moringa, Yucca and Eucalyptus. Also, propolis, as a natural resinous product of bees is widely used in animal and poultry feeds. There are many important bioactive and antimicrobial compounds found in natural feed additives. They have a positive effect on growth performance [7–10], feed efficiency [11–14] immunological status [15–17], reproduction [18–21] and gut microflora [17,22–24] in rabbits. Additionally, incorporating some natural feed additives into rabbit’s diets can help to mitigate the adverse effects of heat stress [25–29]. In the present review, the advantage of using some natural feed additives in rabbit production under high environmental conditions has been described and reviewed.

## MORINGA

The genus Moringa belongs to the Moringaceae family and includes 13 species. The Moringa tree grows in semiarid, arid, tropical, and subtropical areas of Africa, Central and South America, and Southwest and Southeast Asia. It prefers low water and neutral to slightly acidic, sandy, loamy, or sandy-loam soil [10,30,31]. The moringa name is derived from the Tamil word “murungai”, which means “twisted pod,” i.e., young fruit. Moringa oleifera is one of the moringa species. It is the most widely cultivated species. It has fast growth and medical effects, and the leaves have high protein, vitamin A, C, and E, mineral content, as well as carotenoids, flavonoids, polyphenols, and natural antioxidants [5,8,32,33]. The name is derived from the Latin words oleum “oil” and ferre “to bear”. Moringa oleifera, commonly known as the drumstick-tree, or horse radish tree, or miracle tree, or mother’s best friend [34–36].

### Production performance

The present section deals with the effects of M. oleifera (MO) leaf supplementation on productive performance, carcass traits, biochemical parameters, and immunity of rabbits. Moringa oleifera has a high concentration of quality protein, amino acids, vitamin C and E, mineral content, and flavonoids, which can affect rabbit productivity [37,38]. A significant improvement in final body weight, growth performance, and feed conversion ratio (FCR) in New Zealand White (NZW) growing rabbits fed a ration supplemented with 5.5%, 11.0%, and 16.5% MO dry leaves instead of 10%, 20%, and 30% of the protein content of the basal diet [39]. Additionally, El-Desoky et al [19] found significant improvement in body weight, daily gain, and FCR of NZW bunnies fed moringa leaf meal at the end of the fattening period (5 to 13 weeks of age) in favor of the high-level group (6%) followed by the low-level group (3%) and then by the control group. The daily feed intake decreased with using the three moringa leaf meals, with insignificant differences. Similar findings of feed intake and FCR were reported by El-Badawi et al [23], who supplemented NZW rabbits with moringa dry leaves at two levels (0.15% and 0.30%). These results may be due to its important role as a natural growth promoter and having a bacterial probiotic effect resulting from high content of phytochemical compounds. Growing rabbits that received a basal diet supplemented with 30, 60, and 90 mL of aqueous moringa oleifera leaf extract in their drinking water had a significant improvement in final body weight, daily gain, and FCR after an 8-wk-trial [40,41]. A slight increase in the body weight of NZW rabbits fed with moringa oleifera leaf meal (MOLM) at levels of 0%, 3%, and 6% was noticed from 6 to 16 weeks of age [42]. Khalil et al [43] reported that the NZW growing rabbits supplemented with 200 mg MO leaf/kg diet showed the highest body weight and daily gain. Moreover, FCR improved as the supplementation level increased. El-Adawy et al [13] found that there was improvement in body weight, daily gain, daily feed intake, and FCR of NZW growing rabbits at 12 weeks of age when supplemented with dried moringa oleifera leaf protein (1.5% of the diet), and the lowest results were found for control rabbits. Selim et al [8] reported that production performance (body weight gain, and FCR) significantly improved for NZW growing rabbits supplemented with moringa oleifera leaves up to 1.5 g/kg diets. These results may be due to the bioactive compounds in moringa leaves. According to Salem et al [10], the productive performance of Alexandria line rabbits supplemented with MO significantly improved, and the highest weights and gains were found in those supplemented with 20% of MO. They attribute their findings to high levels of amino acids, vitamins (A, B, C, D, K), and macro elements in MO. Similar findings were reported by Jiwuba and Ogbuewu [44]. They found a significant increase in body weight and daily weight gain in rabbits given a diet containing 20% or 30% MOLM. In the hot weather of Saudi Arabia, Aljohani and Abduljawad [45] reported increasing body weight, daily gain, and average daily dry matter intake in NZW rabbits given a diet supplemented with dried MO leaves (500 or 1,000 mg/kg diet). In addition, FCR significantly improved with the increasing levels of MO. These improvements may be because of the high content of amino acids, minerals, and antimicrobial agents in the leaves of moringa. In Nigeria, Abubakar et al [46] reported that the daily gain of rabbits increased as the dietary level of MOLM increased. They added that weaned rabbits could utilize MOLM at up to 45% without harmful effects. On the other hand, Olatunji et al [33] found that there was no significant difference in the final body weight of supplemented growing rabbits with moringa leaf meal at levels of 5%, 10%, 15%, and 20%. In West Africa, Djakalia et al [47] reported that the heaviest body weight and the best growth rate were recorded in rabbits fed a diet supplemented with moringa. In China, Sun et al [48] found an improvement in weight gain and FCR of NZW rabbits given a diet containing 20% rather than 30% of MO leaves. They attributed this result to the high content of phytochemical compounds in the leaves. Yasoob et al [9] studied the effect of the dietary inclusion of MO leaves powder on the productive performance of NZW rabbits under heat stress (35°C for 7 h daily). They found a significant increase in average daily gain, daily feed intake, and FCR in the rabbit supplemented groups.

On the other hand, Badawi et al [36] and Gomaa et al [49] did not detect an improvement in body gain or final body weight of NZW rabbits fed different levels of MOLM. Likewise, El-Badawi et al [5] reported that NZW growing rabbits fed on moringa oleifera did not show a positive result in daily feed intake, average daily gain, and FCR. Under hot environmental conditions, there was no improvement in the daily gain of mixed rabbit breeds supplemented with MOLM at levels of 25% and 50% as a replacement of protein [50]. Similarly, Abiodun and Olubisi [51] found a significant reduction in the weight gain of crossbred rabbit bucks (NZW×Chinchilla) fed a supplemented diet with MOLM. They reported that this negative result may be due to of tannin and saponin content in MOLM. Additionally, in crosses of New Zealand, California, and English rabbit breeds, Hernández-Fuentes et al [52] indicated that body weight and FCR were similar for all treatments (0%, 10%, 20%, and 30% MOLM). Moreover, the daily gain decreased throughout the experimental period in treated rabbits compared with the non-supplemented group. Bakr et al [42] reported that there was no effect of dietary supplementation of MOLM on daily feed intake or FCR in NZW growing rabbits kept under Egyptian conditions.

### Carcass traits

Numerous studies found that including moringa olifera in the diet or drinking water had a positive effect on carcass traits [39,41]. Nuhu [38] found that there were numerical increases in the slaughter weight, hot carcass weight, and dressing percentage in mixed rabbit breeds given moringa olifera leaf meal at levels of 5%, 10%, 15%, and 20%. In addition, the results show that the meat quality significantly improved in groups supplemented with MOLM owing to increased protein content and lower fat level in the meat. El-Badawi et al [23] reported that the carcass traits and meat yield were higher in rabbits fed 0.15% or 0.30% moringa leaf powder than in the control group. Abubakar et al [46] reported that the carcass weight increased with increasing morigna levels (15%, 30%, and 45%) in supplemented diet of weaned rabbits. However, dressing percentage, organ weight, and abdominal fat were not affected by moringa supplementation levels. Omara et al [39] noticed that there was an improvement in weights of slaughter, carcass, head, heart, tests, and dressing percentage, while abdominal fat weight decreased with increasing levels of moringa oleifera supplementation. El-Desoky et al [19] showed that the carcass weight, dressing percentage, and back quarters percentage were significantly higher in rabbits fed a supplemented diet with moringa. Conversely, Gomaa et al [49] found that there were no differences in carcass characteristics among all the treated groups of NZW growing rabbits aged 12 weeks. Additionally, Mos et al [53] reported that the MOLM failed to show a difference in carcass traits of rabbits. Jiwuba and Ogbuewu [44] suggested that rabbits fed MOLM had higher slaughter weight, carcass weight, dressing percentage, hind limb %, liver %, and kidney %. In addition, rabbits fed 20% MOLM showed the highest percentages of loin, fore limb, and thoracic cage. They attributed the increase in cuts to the heavier weight of rabbits as a result of the biological value of MOLM and added that the higher values of liver and kidney are due to the increase in physiological and metabolic activity that removes the toxicity. Selim [8] found that the NZW supplemented with MO leaves showed an increase in dressing percentage, spleen percentage, and intestinal length. In addition, the abdominal fat content decreased as MO leaves increased in diets. They attributed the increment in dressing percentage to the heavier weights of rabbits at slaughter, while the improvement in spleen percentage was attributed to the improving immunity of treated rabbits. Sun et al [48] reported that MO addition had no significant effect on the yield of the carcass. In the hot season of Saudi Arabia, Aljohani and Abduljawad [45] reported that there was a significant improvement in the percentage of carcass, liver, and total edible parts of NZW rabbits supplemented with dried moringa olefira leaves. They attributed these results to the improvement in the metabolism and immunity of treated rabbits. Baker et al [42] reported a significant increase in the empty carcass and total edible part weights of NZW growing rabbits supplemented with 6% MOLM. There were no differences in dressing percentage or weight of the head and edible giblets among treatment groups [42].

### Hematological parameters and blood biochemistry constituents

Many studies determined the change in the blood profile of rabbits due to natural plant supplementation. Aljohani and Abduljawad [45] reported that the level of glucose, urea, and total cholesterol significantly decreased as the dried moringa oleifera leaves level increased in weaning rabbits of NZW. While, aspartate transaminase (AST), alanine transaminase (ALT), alkaline phosphatase (ALP), red blood cell (RBC), white blood cell (WBC), and platelet (PLT) were significantly higher in the treated groups. They added that supplementation with dried moringa olefira leaves up to 1,000 mg/kg diet may improve biochemical parameters and blood constituents, which reflect on the health of rabbits. Sun et al [48] found that albumin, low-density lipoprotein (LDL) cholesterol, T_3_ and T_4_ values, and the activity of superoxide dismutase (SOD) and catalase (CAT) were significantly affected by supplemented moringa oleifera leaves (MOL) in a rabbit’s diet. Khalil et al [43] pointed out that moringa oleifera leaf powder (MOLP) has no harmful effects on protein metabolites (total protein, albumin and globulin), kidney (creatinine and urea), and liver (AST and ALT) functions. They reported that MOLP supplementation at level 200 mg/kg diet had positive effect on hematological parameters and lipid profile.

On the other hand, Debola et al [54] found that packed cell volume (PCV), RBC, haemoglobin, and WBC increased as the moringa-based diet increased, with insignificant differences in supplemented cross-bred rabbits with moringamat 25% and 50% levels. The rabbits fed on a 50% moringa-based diet had higher neutrophils. Lower lympocyte values were found in rabbits fed a 25% moringa-based diet. The lowest values of monocytes, eosinophils, and basophils were found in rabbits fed 50%. Rabbits fed a 50% moringa based diet showed the lowest values of total protein, albumin, and total cholesterol. They concluded that moringa leaf can be added up to a 50% level without any harmful effect on hematological or serum biochemistry. The physiological status improved because of the low total cholesterol. Salem et al [10] pointed out that rabbits supplemented with 20% moringa leaves had the highest hemoglobin, Hb, and PCV because MOL contains vitamins, iron, and protein. They found low values of cholesterol, LDL and malondialdehyde (MDA) and high values of WBCs, globulin, high-density lipoprotein (HDL), and total antioxidants in the blood of rabbits fed MOL.

### Immunity and gut microflora

A recent review illustrated that supplementing rabbits with moringa leaves improved immunity response because moringa leaves have proteins and various peptides. Isitua and Ibeh [35] on adult male Chinchilla rabbits, reported that moringa leaves stimulate B-cells to produce antibodies to improve immunity. Also, there was an increase in CD4 cells, which play an important role in stimulating cell mediated immunity. Sun et al [48] reported that there were high values of liver and spleen indexes in NZW rabbits fed moringa oleifera leaves at levels of 20% and 30%, which means that moringa leaves have a positive effect on immunity in rabbits. Khalil et al [43] reported that the highest immunity status (antibody and titer of lysozyme) was found in growing rabbits fed 200 mg of moringa oleifera leaf powder per kg diet. Selim [8] found that there are high levels of total protein and globulin in the rabbits supplemented with moringa oleifera leaves, which means better immunity. They illustrated that there was a decrease in abdominal fat index in the rabbit supplemented with moringa leaves, which had a positive effect on immunity. Also, in non-tropical area (under winter conditions), Salem et al [10] indicated that increasing the inclusion of moringa levels resulted in a significant increase in lymphocytes, immunoglobulin Y (IgY) and immunoglobulin M (IgM) levels compared to the control rabbit group. Moreover, an increase in CD4 cells and an improvement in cell-mediated immunity were found.

## EUCALYPTUS

The Eucalyptus genus belongs to the Myrtaceae family. Eucalyptus is a general name for up to 700 different species that are found in many regions worldwide, including Australia, China, India, Portugal, Spain, Egypt, Algeria, the southern United States and South America. Eucalyptus is a tall, evergreen tree that is fast growing. The name Eucalyptus is derived from the Greek words, where “eu” means “good or well or true” and “kalypto” means “cover or hide”. That refers to the operculum covering the flower buds. There is a large variation in the chemical composition of the Eucalyptus species. The highest percentages of essential oils in the leaves of Eucalyptus are 1,8-cineol and -pinene, which ranged from 49.07% to 83.59% and 1.27% to 26.35%, respectively [55]. Many investigators reported that Eucalyptus oil and leaves had high values of neutral detergent fiber (NDF), acid detergent fiber (ADF), and lignin [56], p-cymene, 1,8-cineole, β-phellandrene, spathulenol, cryptone aldehydes, cuminal, uncommon and phellandral, α-phellandrene, β-phellandrene leading to multi-functional such as antibacterial, antifungal, anti-inflammatory and antioxidative properties [55,57–59]. Dogan et al [60] reported that essential oil in the leaves of Eucalyptus inhibited the growth of either Gram-positive (*S. aureus* and *B. subtilis*) or Gram-negative (*E. coli* and *Streptococcus* sp.) bacteria strains. These results are related to the increase in feed utilization efficiency and enhanced immunity, which reflect on health and growth [61–63]. Many studies have shown that eucalyptus oil and leaves can be used to improve productive, immunological, and physiological traits in humans [64,65], poultry [59,66,67], and ruminants [68]. The effect of dietary eucalyptus supplementation in rabbits on the productivity, hematological parameters, and biochemicals of blood and immunity traits under high environmental temperatures is scanty.

### Effect on productivity performance

There are inconsistent results on the supplementation of eucalyptus leaves or eucalyptus oil in rabbit performance (body weight, body gain, feed intake). Ahmed et al [69] reported that either body weight or daily gain of rabbits fed diets containing two levels of Eucalyptus leaves failed to show any differences. While rabbits fed a diet with eucalyptus leaves had a higher feed intake value than that of the control group. They attributed the higher feed intake to the improvement of the palatability of diets containing eucalyptus leaves as a result of the content of eucalyptus volatile oil. The results of Kaur et al [70] showed that the crude fiber and crude protein digestibility of laying hens fed a diet supplemented with eucalyptus did not differ from that of the control group. Fathi et al [17] did not detect significant differences between rabbits given a ration supplemented with eucalyptus leaves and the un-supplemented group in body weight or weight gain. The group supplemented with 0.1% eucalyptus showed the highest feed intake, while those supplemented with 0.2% had the lowest feed intake. They found that there was no significant difference due to eucalyptus supplementation on FCR. Different results were found by Waly et al [71] in terms of body gain and feed intake, where they found that body gain of rabbits supplemented with eucalyptus showed greater gain and lower feed intake. They added that FCR was improved as eucalyptus levels increased. These results may be due to the positive effect of eucalyptus on primary antibody responses. In addition, Mohebodini et al [67] on broiler chickens found that there was a linear increase in body weight gain and a reduction in FCR.

### Carcass traits

Body weight at slaughter and carcass traits of NZW rabbits did not differ among groups fed 0, low, and high levels of eucalyptus [69]. The same trend was found for the percentage of heart, kidney, and liver, while inedible parts and full stomach showed a significant increase in groups fed with eucalyptus leaves. On the other hand, Fathi et al [17] reported that the highest dressing percentages, fore part (%) and mid part (%), were found in growing rabbits supplemented with 0.1% eucalyptus, while those supplemented with 0.2% eucalyptus had the lowest ones. They attributed their results to the high tannin level of 0.2%. The opposite result was found for the percent of the hind part, where rabbits fed 0.2 percent eucalyptus had the highest percent, followed by those supplemented with 0.1 percent and then by the control group.

### Hematological parameters and blood biochemistry constituents

The results of Fathi et al [17] on two breeds of rabbit (V-Line and Jabali) pointed out that rabbit supplemented with eucalyptus leave with a level of 0.1% had the highest hemoglobin (HGB), RBCs, and haematocrit without significant difference. The PLTs had the highest value for the rabbits fed 0.20% eucalyptus. On the other hand, Bello [72] in quails and Liu et al [71] in rabbits found that supplementing the diet with eucalyptus significantly increased HGB and RBC. They attributed their results to the iron, beta-carotene, and vitamin C content in eucalyptus. There were different results concerning total protein, serum globulin, and serum albumin. Ahmed et al [69] reported that supplementing diets with eucalyptus showed a significant increase in albumin values while globulin had significantly lower values. Fathi et al [17] found that total protein and serum globulin significantly increased in rabbits fed a diet supplemented with 0.20% eucalyptus. They added that blood cholesterol and triglycerides concentrations were not affected by supplementing with eucalyptus. Ahmed et al [69] found that a diet supplemented with eucalyptus resulted in increasing values of AST, ALT, and alkaline phosphates. They added that adding eucalyptus to the diets of rabbits is considered safe until 13.5 percent. In addition, Waly et al [71] found that there was no effect of supplementation with eucalyptus on the AST and ALT activities in growing rabbits. Fathi et al [17] reported that total antioxidant capacity improved as eucalyptus levels increased in the diets of V-line and Jabali rabbits. This development may be due to the presence of polyphenols, 1,8-cineole, and tannins in eucalyptus, which play a vital role in increasing the antioxidant activity Liu et al [73] on rabbit, and Chen et al [74] on laying hen).

### Immunity and gut microflora

According to the literature, eucalyptus improves immune response due to its tannin content [17,73] and phenolic compounds or essential oils [55,67]. These components play an important role in improving immunity and reducing total bacteria in the gut or cecum. Liu et al [73] reported that there was decreasing MDA in rabbits fed a diet containing tannins. Sebei et al [55] showed that essential oils had antibacterial activity against Listeria and Bacillus. Fathi et al [17] illustrated that the immunity of rabbits increased as eucalyptus levels increased. They added that there was a reduction in total bacterial count, *E. coli*, and *Salmonella* sp. Rabbits fed 0.1% and 0.2% eucalyptus leaves showed the following results: in addition, Mohebodini et al [67] on poultry found that the cecal *E. coli* population was reduced as the eucalyptus level increased, while *Clostridium* spp. and coliforms were not affected.

## YUCCA

*Yucca schidigera* (known as yucca, or the Mojave yucca, or Spanish dagger) is one of the flowering plants and it is one of the species of yucca plants belonging to the family Agaves (Agavaceae). It is well grown in the hot regions of the southwestern United States, the Caribbean islands and Mexico. Yucca grows mostly in the desert and semi-desert areas and needs sun, sandy soil, and good ventilation. Recently, yucca plant extracts have been widely used as natural additives for livestock. The Yucca extract is prepared by drying and grinding the plant. The reviewed publications pointed out that Yucca extract has a high level of saponins, enzymes, and phenolic compounds with antioxidant action [11,22,75–79]. In addition, Svoradová et al [21] and Piacente et al [80] reported that resveratrol, a phytochemical component in yucca, is reasonable for antiviral, antiplatelet, and antioxidant purposes. Therefore, Yucca is used as a natural feed additive to improve feed efficiency, digestibility of nutrients, productivity, and reproductively of livestock animals, rabbits, and poultry [22,77,81–83]. In addition, yucca reduces either ammonia levels in the livestock environment or methane production [11,75,84]. Most of the research studied the effect of yucca supplementation on growth performance, but few investigators studied its effect on carcass traits, blood parameters, and immunity. Many authors pointed out that yucca supplementation had no effect in reducing either ammonia levels (in blood and in houses) or urea in the blood of rabbits.

### Effect on productive performance

Amber et al [85] used yucca extract or probiotic supplementation in the diets of NZW rabbits from 5 to 13 weeks of age. They found that the rabbits fed on yucca showed the highest daily gain, FCR, and digestibility values of dry matter, crude protein, and ether extract. They attributed these improvements to the improvement in the health of rabbits as a result of decreasing ammonia levels and urea in the blood. Results of Abaza and El-Said [75] illustrated that yucca addition improved body weight, weight gain, and FCR of rabbits supplemented with 100 mg of yucca/kg diet. Chrenková et al [22] attributed the improving body weight, weight gain, and feed efficiency to the high level of steroidal saponins and phenolic in yucca extract. Földešiová et al [82] studied the effects of two concentrations of yucca powder added to the diets of rabbits (low, 5 gm/100 kg diet, and high, 20 gm/100 kg diet) on weight gain. They found that the low supplementation had the heaviest body weight and total body weight gain of New Zealand rabbit does aged three months. They pointed out that the improvement may be due to yucca’s rich source of polyphenols, which encourage body weight gain. Földešiová [7] found that supplementation of yucca to NZW rabbit does improve the growth performance because of the effect of plasma levels of progesterone (P4), oxytocin, and prostaglandin F. Ashour et al [11] found that there was an improvement in the FCR of rabbits with a high level of yucca. This may be due to saponins and phenolic materials in yucca extract, which have antimicrobials, antioxidant, and antiviral properties. Results of studies conducted in non-tropical areas, suggested that the dietary supplementation of Yucca schidigera extract can stimulate rabbit growth [7] and fecundity [7,86]. On the other hand, Bergero et al [87] found that yucca supplementation did not show any improvement in body weight, gain, and digestibility of organic matter, energy, and crude protein in a rabbit flock raised in Italy. Ashour et al [11] reported that there was insignificant improvement in body weight and body gain of NZW rabbits supplemented with three levels of yucca extract (200, 400, and 600 mg yucca extract/kg diet), but the rabbits supplemented with 600 mg yucca showed heavier body weight than the control group without any significant differences.

### Carcass traits

Unfortunately, only a few references that deal with the carcass characteristics of rabbits supplemented with Yucca can be found. Abaza and El-Said [75] results of slaughtering rabbits at the end of the experiment indicate that the dressing percentage and abdominal fat significantly decreased as the level of yucca increased in the diet of rabbits. Ashour et al [11] reported that yucca supplementation had a significant effect on carcass yield, dressing percentage and relative weights of kidney, skin, and legs, where the highest results were found in the carcass of rabbits supplemented with the highest level of yucca (600 mg/kg diet). In addition, the treatments failed to show an effect on the relative weights of the heart, spleen, liver, and lung.

### Hematological parameters and blood biochemistry constituents

Amber et al [85] showed that yucca extract decreases the level of ammonia and urea in the blood, which has an impact on the improving health of rabbits. Abaza and El-Said [75] reported that the best levels of globulin, total protein, RBCs, WBCs, and PCV were found in the blood of rabbits supplemented with 100 mg/kg diet Yucca Schidigera powder. There was an insignificant effect of the different treatments on the values of total protein, albumin, and Hb concentration. In addition, they found that urea in the blood decreased as the level of yucca supplementation increased, and they attributed this result to saponins in the yucca and ammonia in the intestines. Ashour et al [11] pointed out that yucca supplementation with levels of 200, 400, and 600 mg/kg diet failed to show a significant effect on total protein, LDL-cholesterol, AST, and ALT, while the treatment had a significant effect on ammonia, triglycerides, total cholesterol, and HDL-cholesterol, where these levels decreased as yucca supplementation increased.

### Immunity and gut microflora

Results of Ashour et al [11] pointed out that yucca supplementation to the basal diet of rabbits had a significant effect on parameters of immunity. They found that yucca supplementation improved immunity where the glutathione peroxidase and catalase activities were affected with a significant effect.

## PROPOLIS

Propolis is one of the phytogenic feed additives, which are considered a product of plant resinous substances collected by honeybees. It has characteristics such as strong antioxidant, anti-inflammatory, and immunomodulation activities [88].

### Characteristics of propolis

The term “propolis” was derived from the Greek language (two Greek words: “pro” and “polis”). The first word, “pro,” means “in defense of”; the other word, “polis,” means the city. Thus, bees used propolis to repair and protect their combs [89–92]. Propolis is sometimes called bee glue [93,94]. Propolis is considered one of the natural feed additives. It is a complex of resinous substances collected by honeybees from different parts of plants, such as buds, flowers, leaf buds, branches, barks, exudates, and wax [95–97]. The main sources of propolis are poplar trees in North America, Europe, Asia, and the northern regions of KSA and Egypt [91,98]; *Baccharis dracunculifolia* leaf in Brazil; *Betula verrucosa* in Russia [99,100]; and *Clusia rosea in Cuba* [98,101,102]. The color of propolis varies depending on its origin; it can be creamy, yellow, green, or light to dark brown [103,104]; and it can have different biological activities [105]. Recently, propolis use has become widespread, especially in temperate zones, for its effects. Propolis has biological activities as antioxidants and antimicrobials. Propolis contains high amounts of phenolic acids and esters, flavonoids, amino acids, vitamins, minerals, and enzymes [106–108]. In addition, propolis has antibacterial effects [109]. Itavo et al [110] reported that propolis affects all gram-positive and some gram-positive bacteria.

The type of plants used by the bees and the season had a strong effect on the chemical composition of propolis [98, 111–113]. Therefore, Araujo et al [114] reported that the propolis in temperate zones differs in its chemical composition. They added that this propolis contains 50% to 60% resins and balsams, 30% to 40% of wax, 5% to 10% of essential and aromatic oils, 5% of pollen, and 5% of other substances. On the other hand, Sforcin [115] noticed that there was no different effect of season on Brazilian propolis composition all year because the propolis was found and collected in the summer season only in the Northern Hemisphere, which is considered a temperate zone. The biological properties of propolis have been reported by numerous authors, and we will illustrate these effects below.

### Effect on productive performance

Because propolis contains antioxidants, vitamins, minerals, phenolic compounds, and enzymes, adding propolis to either diets or drinking water for rabbit feeding increased body weight, weight gain, and FCR. In Egypt, Kamel et al [103] added propolis extract orally to a water suspension containing 100, 200, and 300 mg/kg body weight (BW)/d. They showed that NZW rabbit females treated with propolis increased body weight and reduced feed intake, especially for rabbits treated with the medium dose of propolis (200 mg/kg BW) compared to the control group. The improvement in growth performance may be attributed to different nutritive compounds in propolis. The same results were also found for bunnies treated with propolis, where they had heavier weights and gains from birth to 28 days. Also, Hashem et al [116], on V-line rabbits aged 5 weeks and weighing 586.7 g, received a basal diet supplemented with 150 or 300 mg of propolis/kg dry matter of diet for 5 weeks. They found that rabbits supplemented with propolis at two levels showed significantly higher body weights and weight gain than the control group. The authors attributed the increased weight and gain to the effects of 3,3-dimethyl-2-phenyl-2-(1-oxo-1,2,3,4-tetrahydronaphthalen-2-yl) azirane, which is found to make up 21.40% of propolis. In addition, Attia et al [109] and Mona et al [117], pointed out that there was an increase in body weight, weight gain, feed intake, and FCR in rabbits with propolis. They went on to say that the antioxidants, vitamins, minerals, phenolic constituents, and enzymes found in propolis may be responsible for these effects. The offspring of V-Line rabbits fed a diet supplemented with propolis exhibited improved growth performance at 28 days of age under Saudi Arabia conditions [118]. Also, they supplemented does aged 5 months with propolis orally as a water suspension for three days a week for five weeks (1 week before mating and 4 weeks after mating), and they found that does with propolis had significantly heavier body weights, higher gain, and the lowest feed intake. In addition, under Egyptian summer conditions, Gabr et al [96] found that adding propolis significantly improved the live body weight, daily gain, and FCR of NZW growing rabbits. Additionally, a significant increase in body weight, body weight and FCR was found in NZW rabbits fed a diet supplemented with Egyptian propolis for eight weeks [14]. They attributed their findings to the effect of propolis on the growth of beneficial bacteria in the intestine as well as the stimulation of saccharase, amylase, and phosphatase activities. In Poland, Kupczyński et al [119] noted that adding ethanolic extract of propolis to drinking water for rabbits with chronic diarrhea resulted in reducing the duration of diarrhea and improving final body weight and feed intake. However, there is a difference in many experiments in the effects of using propolis in animal feeding. This difference may be due to the dosage added or chemical composition of propolis. On the other hand, many scientific authors reported that there were no significant effects on the body weight of rabbits as a result of propolis supplementation [97,120,121]. The same trend was found in the case of feed utilization, where Piza et al [97] reported that adding crude propolis did not affect the feed efficiency or diet digestibility in New Zealand rabbits.

### Carcass traits

Many studies found that propolis supplementation had no effect on the carcass traits of fattening rabbits [116,120,122]. Attia et al [109] found that the NZW rabbits supplemented with propolis showed a significantly higher dressing percentage than the control group under summer conditions. The carcass of growing rabbits supplemented by propolis failed to show a significant effect on carcass traits, but there was a higher relative weight of testes and lower body fat weight [96]. Hashem et al [116] reported that the propolis administration did not affect most of the carcass traits except the relative weights of lung and abdominal fat, where the relative weights of lung increased while the relative weight of abdominal fat decreased. In general, they reported that the propolis administration (150 and 300 mg/kg diet) did not affect the relative weight of internal organs in growing rabbits. In Spain, Oliveira et al [123] did not find any effect for adding a green propolis at 0, 50, 100, 150, and 200 mg/kg body weight on the entire carcass traits. Waly et al [14] found that supplementing NZW rabbits with Egyptian propolis increased the percentage of edible parts, the dressing percentage, and decreased internal fat percentages. They added that the organ percentages, such as liver, kidney, and heart, failed to show any effect in supplemented rabbits.

### Hematological parameters and blood biochemistry

Due to flavonoids, steroids, and phenolic acids in propolis, many results indicated that there was an improvement in the health of rabbits fed propolis. Attia et al [118] reported that supplemented V-line rabbits fed orally with propolis have higher values of total protein, albumin, globulin, glucose, and total lipids than control ones. They added that values of globulin/albumin ratio, cholesterol, plasma urea, urea/creatinine ratio, and liver enzymes (AST and ALT) were lower in the treated than in the control group. El-Hanoun et al [124] found the same results and attributed their results to the higher biological activity of propolis to prevent peroxidation of lipids. In addition, Kamel et al [103] supplemented rabbit does with propolis and found high values of plasma total proteins, globulin, and glucose. Also, there was no variation in plasma albumin and creatinine concentrations of doe rabbits treated compared to the control group. A significant decrease was found in total lipids, urea, cholesterol serum, and liver enzyme activity (AST and ALT). They reported that there was an improvement in the functions of the kidney and liver because of a reduction in lipids, urea, and cholesterol. The authors attributed the higher hemoglobin, red blood cell, white blood cell, and packed cell volume to the high iron and selenium in propolis. Similar results were found in growing rabbits supplemented with propolis, where there were decreased values of plasma albumin, total lipids, cholesterol, urea, creatinine, AST, and ALT, while values of plasma globulin were high. The authors said that these effects may be due to the high immunity response of supplemented rabbits. Nassar et al [121] found similar results where liver enzymes had lower values in the treated group with propolis. Also, a low level of serum creatinine and urea was reported. They attributed the reduction results to the propolis compounds derived from flavonoids, steroids, phenolic acids, and their esters. In Spain, serum biochemical profiles did not change in growing rabbits fed a diet supplemented with green propolis compared to the control group [123]. Attia et al [20] illustrated that rabbits fed propolis at 300 mg had significantly higher values of T_4_ than the control group, while values of T_3_ decreased in the plasma of those fed propolis at 150 mg. In addition, the control group had higher AST and ALT levels than those supplemented with propolis or bee pollen. A high level of creatinine was found in the group supplemented with a high level of propolis. Some hematological parameters of alloxan-induced diabetic rabbits fed a diet given Iraqi propolis was studied [125]. The highest values of RBC’s and PCV were recorded in rabbits supplemented with 200 mg of Iraqi propolis/kg body weight. The given propolis may be reduce the negative effect of alloxan [125].

### Immunity and gut microflora

Because of its flavonoids and esters content, propolis extract, or propolis powder has been shown in the literature to improve rabbit immunity. Oršolić and Bašić [126] and Park et al [127] indicated that propolis supplementation increases macrophage activity and interleukin levels, which allow them to produce immunoglobulins. In addition, the specific and nonspecific immune responses improved when ethanolic extract of propolis was administrated in combination with formalized inactivated *Pasteurella multocida* vaccine [121]. Attia et al [109] found that administration of propolis alone was unable to improve immune responses in rabbits, but an improvement occurred when propolis was administered in combination with bee pollen. Braakhuis [128] explained the positive effect of propolis supplementation on the increased synthesis of antibodies from lymphoid organs. He added that the phytochemical components of propolis are considered the source of immunological properties, i.e. phenolic acids, flavonoids, esters, diterpenes, sesquiterpenes, lignans, aromatic aldehydes, alcohols, amino acids, fatty acids, vitamins and minerals.

## CONCLUSION

The routine use of antibiotics in intensive farming comes into force due to harmful residual effects on human health. The use of natural feed additives is gaining concern in livestock production. Using some plant extracts or natural products as alternative feed additives has beneficial effects on growth performance and physiological stimulants, as well as for health enhancement. Most of them improve nutrient utilization and absorption by enhancing digestibility. Additionally, the activities of antimicrobial, anti-inflammatory, antioxidant, immune-stimulant, and stabilizing beneficial microflora are also improved, particularly in farm animals suffering from heat stress. The beneficial effects of using moringa, propolis, yucca, and eucalyptus have been intensively reviewed and discussed in rabbits raised either in hot or temperate environmental conditions. Table 1 summarizes the effect of some natural feed additives on the rabbit’s performance and productivity under different environmental conditions.

## Figures and Tables

**Table 1:** Summary of the effect of natural feed additives on rabbit performance under hot climatic conditions

Effect	Ambient temperature/region	Country	Breed	Ref
	**Moringa**			
Growth performance (+), feed intake (+), FCR (+), carcass traits (+)	Hot climate	Egypt	NZW	[39]
			NZW	[19]
			NZW	[23]
			APRI	[41]
Growth performance (+), feed intake (−), FCR (+), carcass traits (+), blood parameters (−)	Hot climate	Egypt	NZW	[42]
Growth performance (+), feed intake (+), FCR (+), carcass traits (+), blood parameters (+), immunity (+)	20°C to 22°C	Egypt	NZW	[43]
			NZW	[13]
			NZW	[8]
			Alexandria line	[10]
Growth performance (+), feed intake (+), FCR (+), blood parameters (+), immunity (+)	Hot climate	Egypt	NZW	[43]
Growth performance (+), feed intake (+), FCR (+)	Hot climate	Egypt	NZW	[13]
Growth performance (+), feed intake (+), FCR (+), carcass traits (+), immunity (+)	Hot climate	Egypt	NZW	[8]
Growth performance (+), feed intake (+), FCR (+), blood parameters (+), immunity (+)	Hot climate	Egypt	Alexandria line	[10]
Growth performance (+), feed intake (+), FCR (+), carcass traits (+), blood parameters (+)	Hot area	KSA	NZW	[45]
Body gain (+), carcass weight (+), dressing % (−), organs weight (−)	Tropical area	Nigeria		[46]
Growth performance (+), feed intake (+), FCR (+), carcass traits (+)	Tropical area	Nigeria		[44]
Growth performance (+), blood parameters (−)	Tropical area	Nigeria		[33]
Growth performance (+)	Semi-arid	West Africa		[47]
Growth performance (+), carcass traits (−), blood parameters (+), immunity (+)	Hot condition	China	NZW	[48]
Growth performance (+), feed intake (+), FCR (+)	35°C	China	NZW	[9]
Growth performance (−), feed intake (+), FCR (+), blood parameters (+), immunity (+)	Hot climate	Egypt	NZW	[36]
Growth performance (−), feed intake (−), FCR (−), carcass traits (−), blood parameters (−)	NA	Egypt	NZW	[49]
Growth performance (−), feed intake (−), FCR (+), caecal microbial (+)	33.1°C	Egypt	NZW	[5]
Growth performance (−), feed intake (+), FCR (+), carcass traits (+), blood parameters (+)	19°C to 29°C	Cameroon	mixed breeds	[50]
Growth performance (−), carcass traits (−)	NA	Nigeria	New Zealand white × Chinchilla	[51]
Growth performance (−), feed intake (−), FCR (+), carcass traits (+), blood parameters (−)	NA	Mexico	New Zealand, California, and English Pot	[52]
Growth performance (+), feed intake (−), FCR (−), carcass traits (+), blood parameters (−)	26.1°C to 28.9°C		mixed breeds	[38]
blood (+)	19°C to 29°C		Cross breed	[54]
Growth performance (−), blood parameters (−), immunity (+)	Tropical area	Nigeria	Chinchilla	[35]
	**Eucalyptus**			
Growth performance (−), feed intake (−), carcass traits (−), blood parameters (+), immunity (+)	NA	Egypt	NZW	[69]
Growth performance (−), feed intake (−), FCR (−), carcass traits (+), blood parameters (+), immunity (+), caecal microbial (+)	24°C to 39°C	Egypt	V-line & Jabali	[17]
Growth performance (+), feed intake (+), FCR (+), blood parameters (+)	Hot climate	Egypt	NZW	[71]
	**Yucca**			
Growth performance (+), FCR (+), blood (+), microbial (+)	Hot climate	Egypt	NZW	[85]
Growth performance (+), FCR (+), carcass traits (+), blood parameters (+)	Hot climate	Egypt, alex	NZWxBB	[75]
Growth performance (+)	17°C±3°C	Slovak Republic	NZW	[82]
Growth performance (+), blood parameters (+)	NA	Slovak Republic	NZW	[7]
Growth performance (+), feed intake (−), FCR (+), carcass traits (+), blood parameters (+), immunity (+)	Hot climate	Egypt zagazig	NZW	[11]
Growth performance (−), feed intake (−), FCR (−)	NA	Torino – Italy	NZW	[86]
	**Propolis**			
Growth performance (+), feed intake (+), FCR (+), blood parameters (+), immunity (+)	Hot climate	Alex-Egypt	NZW	[102]
Growth performance (+), carcass traits (−), blood parameters (+), immunity (+)	23°C to 27°C	Alex-	V-line	[115]
Growth performance (+), feed intake (+), FCR (+), blood parameters (+)	Hot climate	Egypt	NZW	[116]
Growth performance (+), feed intake (+), FCR (+), carcass traits (+), blood parameters (+), immunity (−)	15.1°C to 29.2°C	Egypt	NZW	[108]
Growth performance (+), feed intake (+), FCR (+), carcass traits (+), blood parameters (+), immunity (+)	Hot temperature		broiler chickens	[117]
Growth performance (+), feed intake (+), blood parameters (+)	15.1°C to 29.2°C		V-line	[118]
Growth performance (+), feed intake (+), FCR (+), carcass traits (−), blood parameters (−)	25.1°C to 33.8°C	Egypt	NZW	[95]
Growth performance (+), feed intake (+), FCR (+), carcass traits (+), blood parameters (+), immunity (+)	Hot climate	Egypt	NZW	[14]
Growth performance (+), feed intake (+), FCR (+), blood parameters (+)	20°C±2°C	Poland	Hyplus	[119]
Growth performance (−), carcass traits (+)	NA		NZW	[120]
Growth performance (−), blood parameters (+), immunity (+)	Hot climate	Egypt	NZW	[121]
Growth performance (−), feed intake (−), FCR (−)	23°C	Minas Gerais, Brazil	NZW	[96]
Growth performance (−), carcass traits (−), blood parameters (−)	Cold area	Spain	half-breed rabbits	[123]
Growth performance (+), blood parameters (+), immunity (+)	Hot climate	Egypt	NZW	[20]
Blood parameters (+), immunity (+)	Hot climate	Iraq	Local breed	[125]

NA, not available; FCR, feed conversion ratio.

Plus sign stands for trait improvement and minus sign stands for trait deterioration.
